# Impact of oral statin therapy on clinical outcomes in patients with cT1 breast cancer

**DOI:** 10.1186/s12885-023-10631-w

**Published:** 2023-03-09

**Authors:** Koji Takada, Shinichiro Kashiwagi, Nozomi Iimori, Rika Kouhashi, Akimichi Yabumoto, Wataru Goto, Yuka Asano, Yukie Tauchi, Tamami Morisaki, Kana Ogisawa, Masatsune Shibutani, Hiroaki Tanaka, Kiyoshi Maeda

**Affiliations:** 1 Department of Breast Surgical Oncology, Osaka Metropolitan University Graduate School of Medicine, 1-4-3 Asahi-machi, Abeno-ku, Osaka, 545-8585 Japan; 2 Department of Gastroenterological Surgery, Osaka Metropolitan University Graduate School of Medicine, 1-4-3 Asahi-machi, Abeno-ku, Osaka, 545-8585 Japan

**Keywords:** breast cancer, statin, hyperlipidemia, axillary lymph node, prognosis

## Abstract

**Purpose:**

A previous meta-analysis examining the relationship between statin use and breast cancer reported that the inhibitory effect of statins on breast cancer may be more pronounced in early-stage cases. In this study, we aimed to investigate the effects of hyperlipidemia treatment at the time of breast cancer diagnosis and to examine its correlation with metastasis to axillary lymph nodes among patients with so-called cT1 breast cancer whose primary lesion was 2 cm or less and was pathologically evaluated by sentinel lymph node biopsy or axillary lymph node dissection. We also investigated the effects of hyperlipidemic drugs on the prognosis of patients with early-stage breast cancer.

**Methods:**

After excluding cases that did not meet the criteria, we analyzed data from 719 patients who were diagnosed with breast cancer, with a primary lesion of 2 cm or less identified by preoperative imaging, and who underwent surgery without preoperative chemotherapy.

**Results:**

Regarding hyperlipidemia drugs, no correlation was found between statin use and lymph node metastasis (*p* = 0.226), although a correlation was found between lipophilic statin use and lymph node metastasis (*p* = 0.042). Also, the disease-free survival periods were prolonged following treatment of hyperlipidemia (*p* = 0.047, hazard ratio: 0.399) and statin administration (*p* = 0.028, hazard ratio: 0.328).

**Conclusion:**

In cT1 breast cancer, the results suggest that oral statin therapy may contribute to favorable outcomes.

**Supplementary Information:**

The online version contains supplementary material available at 10.1186/s12885-023-10631-w.

## Background

Although various orally administered drugs are clinically used in the treatment of a wide variety of diseases, it has been reported that some may have unexpected effects on cancer. For example, in a systematic review and meta-analysis, the diabetes drug metformin reduced the risk of colorectal cancer and prostate cancer [1]. However, the analysis in that report did not reveal a reduction in breast cancer risk, whereas some studies have reported that metformin reduces breast cancer risk and improves prognosis [1, 2]. Another meta-analysis of observational studies reported that long-term use of angiotensin-receptor blockers / angiotensin-converting enzyme inhibitors for the treatment of hypertension might reduce the risk of breast cancer [3]. Among these drugs, statins, which are typically used for the treatment of hyperlipidemia, have also been reported to suppress the development of cancer and reduce the rate of recurrence [4–12]. These outcomes may be explained by many preclinical studies that have reported antiproliferative and anti-apoptotic effects in breast cancer [13–17]. In addition, based on the anti-invasive properties [18–22] and metastasis-suppressing effects of statins that have been demonstrated in preclinical studies, some reports have clinically examined their progression-suppressing effects in breast cancer [23–29].

Another meta-analysis examining the relationship between statin use and breast cancer reported that the inhibitory effect of statins on breast cancer may be more pronounced in patients with early-stage breast cancer [30]. Therefore, we hypothesized that statins may affect metastasis to lymph nodes in breast cancer cases involving a small primary lesion. In recent years, axillary surgery for early-stage breast cancer has been reduced due to the increased effectiveness of multidisciplinary treatment before and after surgery, and evaluation of axillary lymph node metastasis before treatment has become even more important. If our hypothesis is correct, statin administration may affect the evaluation. In this study, we aimed to investigate the treatment of hyperlipidemia at the time of breast cancer diagnosis and to examine its correlation with the metastatic status in axillary lymph nodes among patients with so-called cT1 breast cancer involving a primary lesion of 2 cm or less who underwent pathological evaluations of metastasis in an axillary lymph node by sentinel lymph node biopsy or axillary lymph node dissection. We also aimed to investigate the effects of hyperlipidemic drugs on the prognosis of patients with early-stage breast cancer.

## Methods

### Patient background and classification

Seven hundred forty-two patients were diagnosed with breast cancer involving a primary lesion of 2 cm or less by preoperative imaging and underwent surgery without preoperative chemotherapy from April 2007 to March 2020 at Osaka City University Hospital. Pathological diagnosis of breast cancer was based on core needle biopsy (CNB) or vacuum-assisted biopsy (VAB). As an evaluation of their general condition before initiating treatment for breast cancer, the patients were confirmed to have a history of pre-treatment and oral medication use. We classified the drugs used to treat hyperlipidemia for further examination. The pharmacological classification of statins based on their hydrophilicity and lipophilicity was performed according to the classification system widely used in cardiovascular studies [31, 32]. Specifically, rosuvastatin and pravastatin are classified as hydrophilic statins, while atorvastatin, pitavastatin, simvastatin and fluvastatin are classified as lipophilic statins. Either mastectomy or breast-conserving surgery was performed because the preoperative imaging examinations such as ultrasonography (US), computed tomography (CT), and bone scintigraphy revealed that radical resection was possible. Axillary lymph node dissection was performed for cases in which axillary lymph node metastasis was suspected, and sentinel lymph node biopsy was performed for cases in which no metastasis was diagnosed. During surgery for breast cancer, the sentinel lymph node was identified using a combination of radioisotope and dye methods according to previous reports [33, 34]. Histopathological diagnosis of sentinel lymph node metastasis was conducted by slicing the entire sentinel lymph node into 2-mm-thick sections [35, 36]. Sentinel lymph node metastases were categorized by size according to previously reported parameters (macrometastasis: tumor diameter > 2 mm; micrometastasis: tumor diameter > 0.2 mm, ≤ 2 mm or < 200 tumor cells; for isolated tumor cells: tumor diameter < 0.2 mm or < 200 tumor cells) [37]. Axillary dissection was additionally performed in patients with macrometastasis that was confirmed via sentinel lymph node biopsy.

The expression levels of estrogen receptor (ER), progesterone receptor (PgR), human epidermal growth factor receptor 2 (HER2), and Ki67, a marker of proliferation, were examined immunohistochemically in both the biopsy tissue used for breast cancer diagnosis and the surgically removed tissue. Based on the results of the immunohistological staining, breast cancer was classified into the following three subtypes: triple-negative breast cancer (TNBC; negative for ER, PgR, and HER2); hormone receptor (HR)-HER2+ breast cancer (HR-negative and HER2-positive breast cancer; ER-, PgR-, and HER2+); and HR+ breast cancer (hormone receptor-positive breast cancer; ER+ and/or PgR+).

There were 742 preoperatively diagnosed cases of cT1 breast cancer. However, 15 cases did not undergo axillary lymph node dissection or sentinel lymph node biopsy, and eight cases were being treated with unknown medications at the time of diagnosis. Therefore, these 23 cases were excluded from this study, and data was analyzed from the remaining 719 cases.

### Statistical analysis

All statistical analyses were performed using the JMP software package (SAS, Tokyo, Japan). Each correlation was examined using Pearson’s chi-square test. The odds ratio (OR) and 95% confidence interval (CI) were calculated by logistic analysis, and multivariable analysis was performed using the multivariable logistic regression model. Prognostic analyses, such as the calculation of recurrence-free survival (RFS) or overall survival (OS), were conducted using the Kaplan–Meier method and the log-rank test. The hazard ratios (HR) and 95% CI were calculated using the Cox proportional hazards model. Multivariable analysis was performed using the Cox regression model. A *p*-value of < 0.05 was considered statistically significant.

## Results

### Clinicopathological features

Table 1 shows the clinicopathological features of the 719 patients with cT1 breast cancer who underwent surgery without receiving preoperative chemotherapy. The median age was 58 years (range, 29–79 years), and the median tumor diameter was 13 mm (range, 3.0–20.0 mm). A total of 612 patients (85.7%) were positive for ER, 398 patients (55.4%) were positive for PgR, and 621 patients (86.3%) were classified as having HR+ breast cancer, which represented the majority of cases. There were 66 patients (9.2%) with HER2-positive breast cancer, but only 27 patients (3.8%) were classified as having HR-HER2+ breast cancer. Seventy-one patients (9.9%) were classified as having TNBC. Ki67 was expressed at a level higher than 20% in 133 patients (18.5%).

**Table 1:** Clinicopathological features of 719 cT1 breast cancer patients who underwent surgery without preoperative chemotherapy

Parameters	Number of patients (*n* = 719) (%)
Age at operation (years old)	median 58 (range, 29–91)
Tumor size (mm)	median 13 (range, 3–20)
Estrogen receptor	
Negative / Positive	107 (14.9%) / 612 (85.1%)
Progesterone receptor	
Negative / Positive	321 (44.6%) / 398 (55.4%)
HER2	
Negative / Positive	653 (90.8%) / 66 (9.2%)
Ki67	
≤ 20% / > 20%	586 (81.5%) / 133 (18.5%)
Intrinsic subtype	
HR + BC / HR-HER2 + BC / TNBC	621 (86.3%) / 27 (3.8%) / 71 (9.9%)
Pathological axillary lymph node metastasis	
No metastasis / only isolated tumor cell / only micrometastasis / metastasis	573 (79.7%) / 5 (0.7%) / 29 (4.0%) / 112 (15.6%)
Lymph vascular invasion	
No / Yes	528 (73.4%) / 191 (26.6%)
Hyperlipidemia	
No / Yes	572 (79.6%) / 147 (20.4%)
Number of medicine types for hyperlipidemia	
0 / 1 / 2	572 (79.6%) / 139 (19.3%) / 8 (1.1%)
Statins	
Non-user / User	587 (81.6%) / 132 (18.4%)
Lipophilic statins	
Non-user / User	658 (91.5%) / 61 (8.5%)
Hydrophilic statins	
Non-user / User	648 (90.1%) / 71 (9.9%)
Fibrate	
Non-user / User	709 (98.6%) / 10 (1.4%)
Nicotinic acid (tocopherol acetate)	
Non-user / User	712 (99.0%) / 7 (1.0%)
Sterol absorption inhibitors (ezetimibe)	
Non-user / User	713 (99.2%) / 6 (0.8%)

*HER2* Human epidermal growth factor receptor 2, *HR + BC* Hormone receptor-positive breast cancer (ER+ and/or PgR+), *HR-HER2 + BC* Human epidermal growth factor receptor 2-enriched breast cancer (ER-, PgR-, and HER2+), *TNBC* Triple negative breast cancer (ER-, PgR-, and HER2-)

Postoperative pathological examinations revealed no axillary lymph node metastasis in 607 patients (84.4%), including five patients (0.7%) with isolated tumor cells and 29 patients (4.0%) with micrometastases based on sentinel lymph node biopsies. The median number of lymph node metastases in 112 patients (15.6%) with axillary lymph node metastases was two (range, 1–26). Lymphovascular invasion was detected in 191 patients (26.6%).

At the time of breast cancer diagnosis, 147 patients (20.4%) were undergoing treatment with orally administered drugs for hyperlipidemia. Among them, only eight patients (1.1%) were taking multiple drugs, whereas most were treated with single drugs. Among the 132 patients (18.4%) who were being treated with statins, 61 patients (8.5%) were taking lipophilic statins, and 71 patients (9.9%), about half, were taking hydrophilic statins. Specifically, rosuvastatin, one of the hydrophilic statins, users were 36 patients (5.0%) and pravastatin users were 35 patients (4.9%). On the other hand, the results for lipophilic statins were as follows: atorvastatin; 27 patients (3.8%), pitavastatin; 20 patients (2.8%), simvastatin; 13 patients (1.8%), and fluvastatin 1 patients (0.1%). There were 10 fibrate users (1.4%), seven nicotinic acid (tocopherol acetate) users (1.0%), and six sterol absorption inhibitors (ezetimibe) users (0.8%).

### Correlations between clinicopathological features and axillary lymph node metastasis

The correlations between clinicopathological features and axillary lymph node metastasis are listed in Table 2. Metastasis occurred significantly more frequently when the breast cancer tumor diameter exceeded 10 mm (*p* < 0.001). Although the relationship was not statistically significant, metastases tended to be found in PgR-positive breast cancer cases (*p* = 0.063). Metastases occurred significantly more frequently in breast cancer cases involving lymphovascular invasion (*p* < 0.001). Regarding hyperlipidemia drugs, no correlation was found between statin use in general and lymph node metastasis (*p* = 0.226); however, a significant correlation was found between the use of *lipophilic* statins and lymph node metastasis (*p* = 0.042).

**Table 2:** Correlation between axillary lymph node metastasis and clinicopathological features

Parameters	Axillary lymph node metastasis		*p* value
	No metastasis, including even micrometasis (*n* = 607)	metastasis (*n* = 112)	
Age at operation (years old)			0.872
≤ 60	331 (54.5%)	62 (55.4%)	
> 60	276 (45.5%)	50 (44.6%)	
Tumor size (mm)			< 0.001
≤ 10.0	202 (33.3%)	15 (13.4%)	
> 10.0	405 (66.7%)	97 (86.6%)	
Estrogen receptor			0.441
Negative	93 (15.3%)	14 (12.5%)	
Positive	514 (84.7%)	98 (87.5%)	
Progesterone receptor			0.063
Negative	280 (46.1%)	41 (36.6%)	
Positive	327 (53.9%)	71 (63.4%)	
HER2			0.920
Negative	551 (90.8%)	102 (91.1%)	
Positive	56 (9.2%)	10 (8.9%)	
Ki67			0.734
≤ 20%	496 (81.7%)	90 (80.4%)	
> 20%	111 (18.3%)	22 (19.6%)	
Intrinsic subtype HR + BC			0.201
No	87 (14.3%)	11 (9.8%)	
Yes	520 (85.7%)	101 (90.2%)	
Intrinsic subtype HR-HER2 + BC			0.911
No	584 (96.2%)	108 (96.4%)	
Yes	23 (3.8%)	4 (3.6%)	
Intrinsic subtype TNBC			0.162
No	543 (89.5%)	105 (93.8%)	
Yes	64 (10.5%)	7 (6.3%)	
Lymph vascular invasion			< 0.001
No	478 (78.7%)	50 (44.6%)	
Yes	129 (21.3%)	62 (55.4%)	
Hyperlipidemia			0.212
No	478 (78.7%)	94 (83.9%)	
Yes	129 (21.3%)	18 (16.1%)	
Multiple medicine types for hyperlipidemia			0.460
No	601 (99.0%)	110 (98.2%)	
Yes	6 (1.0%)	2 (1.8%)	
Statins			0.226
Non-user	491 (80.9%)	96 (85.7%)	
User	116 (19.1%)	16 (14.3%)	
Lipophilic statins			0.042
Non-user	550 (90.6%)	108 (96.4%)	
User	57 (9.4%)	4 (3.6%)	
Hydrophilic statins			0.746
Non-user	548 (90.3%)	100 (89.3%)	
User	59 (9.7%)	12 (10.7%)	
Fibrate			0.624
Non-user	598 (98.5%)	111 (99.1%)	
User	9 (1.5%)	1 (0.9%)	
Nicotinic acid (tocopherol acetate)			0.341
Non-user	602 (99.2%)	110 (98.2%)	
User	5 (0.8%)	2 (1.8%)	
Sterol absorption inhibitors (ezetimibe)			0.941
Non-user	602 (99.2%)	111 (99.1%)	
User	5 (0.8%)	1 (0.9%)	

*HER2* Human epidermal growth factor receptor 2, *HR + BC* Hormone receptor-positive breast cancer (ER+ and/or PgR+), *HR-HER2 + BC* Human epidermal growth factor receptor 2-enriched breast cancer (ER-, PgR-, and HER2+), *TNBC* Triple negative breast cancer (ER-, PgR-, and HER2-)

Examination of the correlation between lipophilic statin use and clinicopathological factors revealed that the users were significantly older than the non-users (*p* < 0.001) (Table 3).

**Table 3:** Correlation between lipophilic statins user and clinicopathological features

Parameters	Lipophilic statins		*p* value
	Non-user (*n* = 658)	User (*n* = 61)	
Age at operation (years old)			< 0.001
≤ 60	388 (59.0%)	5 (8.2%)	
> 60	270 (41.0%)	56 (91.8%)	
Tumor size (mm)			0.450
≤ 10.0	196 (29.8%)	21 (34.4%)	
> 10.0	462 (70.2%)	40 (65.6%)	
Estrogen receptor			0.247
Negative	101 (15.3%)	6 (9.8%)	
Positive	557 (84.7%)	55 (90.2%)	
Progesterone receptor			0.548
Negative	296 (45.0%)	25 (41.0%)	
Positive	362 (55.0%)	36 (59.0%)	
HER2			0.228
Negative	595 (90.4%)	58 (95.1%)	
Positive	63 (9.6%)	3 (4.9%)	
Ki67			0.554
≤ 20%	538 (81.8%)	48 (78.7%)	
> 20%	120 (18.2%)	13 (21.3%)	
Intrinsic subtype HR + BC			0.367
No	92 (14.0%)	6 (9.8%)	
Yes	566 (86.0%)	55 (90.2%)	
Intrinsic subtype HR-HER2 + BC			0.107
No	631 (95.9%)	61 (100.0%)	
Yes	27 (4.1%)	0 (0.0%)	
Intrinsic subtype TNBC			0.992
No	593 (90.1%)	55 (90.2%)	
Yes	65 (9.9%)	6 (9.8%)	
Lymph vascular invasion			0.504
No	481 (73.1%)	47 (77.0%)	
Yes	177 (26.9%)	14 (23.0%)	
Hyperlipidemia			< 0.001
No	572 (86.9%)	0 (0.0%)	
Yes	86 (13.1%)	61 (100.0%)	
Multiple medicine types for hyperlipidemia			0.003
No	653 (99.2%)	58 (95.1%)	
Yes	5 (0.8%)	3 (4.9%)	
Hydrophilic statins			0.007
Non-user	587 (89.2%)	61 (100.0%)	
User	71 (10.8%)	0 (0.0%)	
Fibrate			0.862
Non-user	649 (98.6%)	60 (98.4%)	
User	9 (1.4%)	1 (1.6%)	
Nicotinic acid (tocopherol acetate)			0.055
Non-user	653 (99.2%)	59 (96.7%)	
User	5 (0.8%)	2 (3.3%)	
Sterol absorption inhibitors (ezetimibe)			0.454
Non-user	652 (99.1%)	61 (100.0%)	
User	6 (0.9%)	0 (0.0%)	

*HER2* Human epidermal growth factor receptor 2. *HR + BC* Hormone receptor-positive breast cancer (ER+ and/or PgR+). *HR-HER2 + BC* Human epidermal growth factor receptor 2-enriched breast cancer (ER-, PgR-, and HER2+), *TNBC* Triple negative breast cancer (ER-, PgR-, and HER2-)

**Fig. 1:**
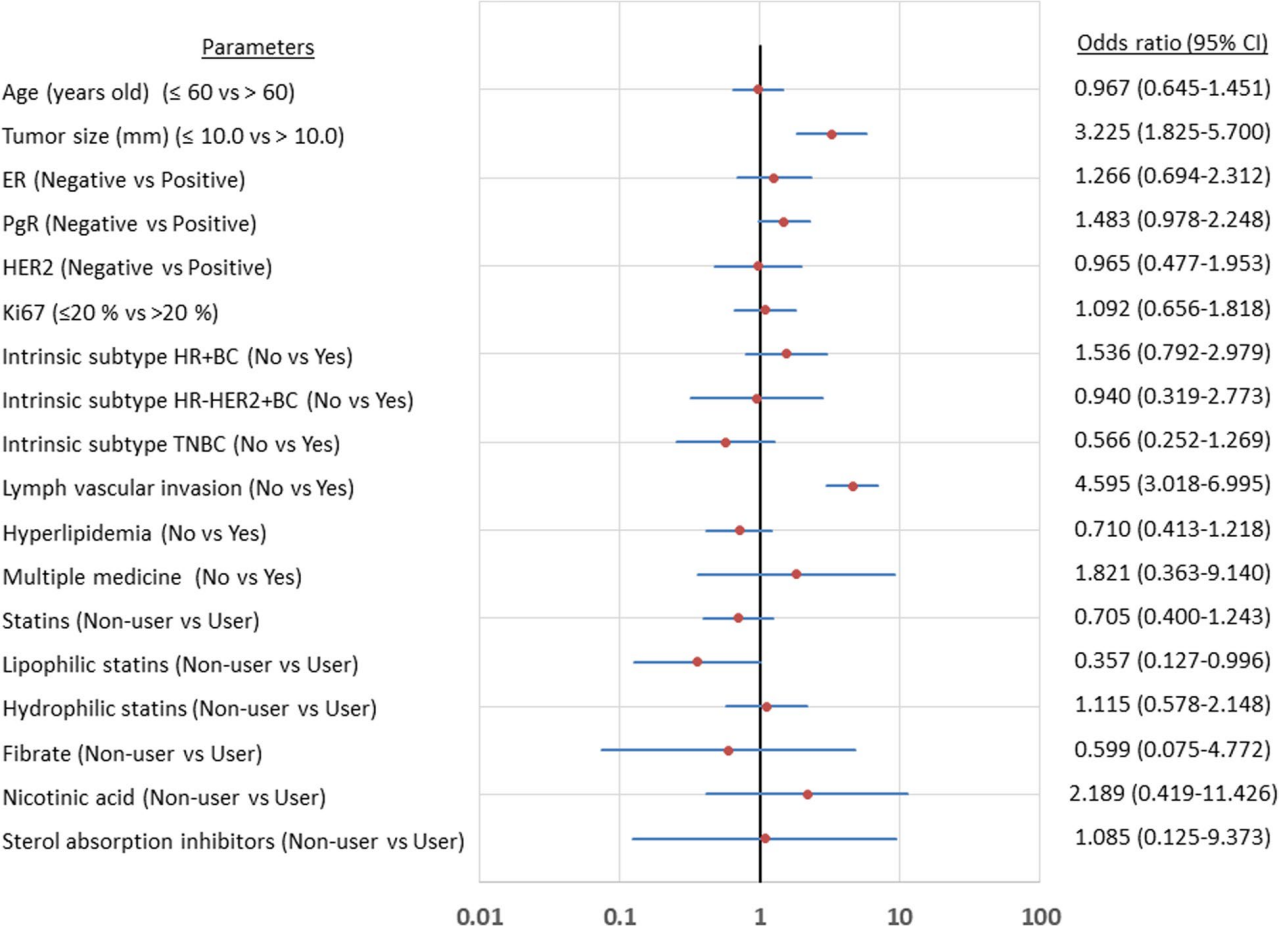
Forest plot showed odd ratios for the univariate association of the risk factors for axillary lymph node metastasis

We examined the factors causing axillary lymph node metastasis in patients with cT1 breast cancer; tumor size (*p* < 0.001, OR = 3.225) and lymphovascular invasion (*p* < 0.001, OR = 4.595), as well as the use of lipophilic statins (*p* = 0.042, OR = 0.357) were the factors associated with axillary lymph node metastasis (Table 4) (Fig. 1). Even after performing the multivariate analysis, these remained independent factors (tumor size: *p* = 0.003, OR = 2.352; lymphovascular invasion: *p* < 0.001, OR = 3.891; lipophilic statin use: *p* = 0.048, OR = 0.384). Thus, lipophilic statin was the only factor that reduced axillary lymph node metastasis.

**Table 4:** Univariate and multivariate analysis with axillary lymph node metastasis for cT1 breast cancer

Parameters	Univarite analysis			Multivarite analysis		
	Odds ratio	95% CI	*p* value	Odds ratio	95% CI	*p* value
Age at operation (years old)						
≤ 60 vs > 60	0.967	0.645–1.451	0.872			
Tumor size (mm)						
≤ 10.0 vs > 10.0	3.225	1.825–5.700	< 0.001	2.352	1.337–4.391	0.003
Estrogen receptor						
Negative vs Positive	1.266	0.694–2.312	0.441			
Progesterone receptor						
Negative vs Positive	1.483	0.978–2.248	0.063	1.457	0.945–2.269	0.089
HER2						
Negative vs Positive	0.965	0.477–1.953	0.920			
Ki67						
≤ 20% vs > 20%	1.092	0.656–1.818	0.734			
Intrinsic subtype HR + BC						
No vs Yes	1.536	0.792–2.979	0.201			
Intrinsic subtype HR-HER2 + BC						
No vs Yes	0.940	0.319–2.773	0.911			
Intrinsic subtype TNBC						
No vs Yes	0.566	0.252–1.269	0.162			
Lymph vascular invasion						
No vs Yes	4.595	3.018–6.995	< 0.001	3.891	2.529–6.016	< 0.001
Hyperlipidemia						
No vs Yes	0.710	0.413–1.218	0.212			
Multiple medicine types for hyperlipidemia						
No vs Yes	1.821	0.363–9.140	0.460			
Statins						
Non-user vs User	0.705	0.400–1.243	0.226			
Lipophilic statins						
Non-user vs User	0.357	0.127–0.996	0.042	0.384	0.113–0.987	0.048
Hydrophilic statins						
Non-user vs User	1.115	0.578–2.148	0.746			
Fibrate						
Non-user vs User	0.599	0.075–4.772	0.624			
Nicotinic acid (tocopherol acetate)						
Non-user vs User	2.189	0.419–11.426	0.341			
Sterol absorption inhibitors (ezetimibe)						
Non-user vs User	1.085	0.125–9.373	0.941			

*HER2* Human epidermal growth factor receptor 2, *HR + BC* Hormone receptor-positive breast cancer (ER+ and/or PgR+), *HR-HER2 + BC* Human epidermal growth factor receptor 2-enriched breast cancer (ER-, PgR-, and HER2+), *TNBC* Triple negative breast cancer (ER-, PgR-, and HER2-), *CI* Confidence intervals

### Effects of lipophilic statins on prognosis

**Fig. 2:**
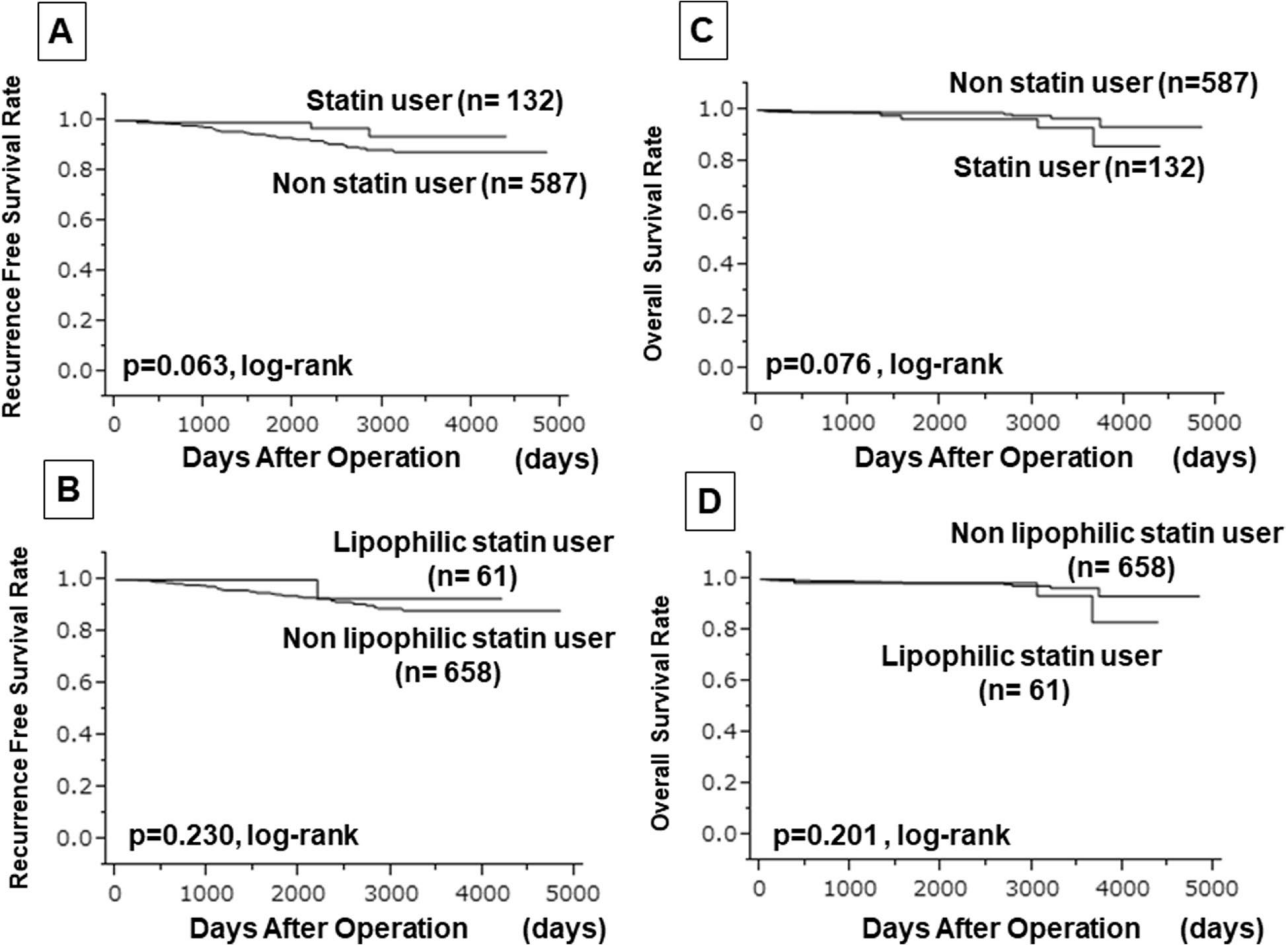
Kaplan–Meier method comparing recurrence-free survival (RFS) and overall survival (OS) by statin or *lipophilic* statin. There was no significant difference in RFS due to statin (**A**) and lipophilic statin (**B**). No significant difference was found in OS due to statin (**C**) and lipophilic statin (**D**)

We examined the prognosis of 719 patients with cT1 breast cancer included in this study. The median follow-up period was 1838 days (range, 54–4841 days). During that period, 42 patients (5.8%) experienced recurrence, three patients (0.4%) died from breast cancer, and 11 patients (1.5%) died from other causes. Univariate analysis of disease-free survival (DFS) times showed that tumor size affected prognosis (*p* = 0.011, HR: 2.902) and that vascular infiltration tended to lead to a poor prognosis (*p* = 0.086, HR: 1.712) (Online Resource Supplementary Table 1). Among the factors, the treatment of hyperlipidemia (*p* = 0.047, HR: 0.399) and statin use (*p* = 0.028, HR: 0.328) were associated with prolonged DFS periods. In the multivariate analysis, only tumor size was an independent factor (*p* = 0.025, HR: 2.620). Similarly, in the univariate analysis for RFS, tumor size (*p* = 0.017, HR: 2.732) as well as statin use (*p* = 0.038, HR: 0.345) affected prognosis (Table 5) (Fig. 2). No clinicopathological factors significantly affected OS (Table 6).

**Table 5:** Univariate and multivariate analysis with recurrence-free survival for cT1 breast cancer

Parameters	Univarite analysis			Multivarite analysis		
	Hazard ratio	95% CI	*p* value	Hazard ratio	95% CI	*p* value
Age at operation (years old)						
≤ 60 vs > 60	0.607	0.310–1.133	0.119			
Tumor size (mm)						
≤ 10.0 vs > 10.0	2.732	1.177–7.946	0.017	2.658	1.14–7.739	0.021
Estrogen receptor						
Negative vs Positive	1.166	0.549–2.870	0.707			
Progesterone receptor						
Negative vs Positive	1.509	0.808–2.950	0.200			
HER2						
Negative vs Positive	0.887	0.214–2.449	0.839			
Ki67						
≤ 20% vs > 20%	0.798	0.274–1.854	0.626			
Intrinsic subtype HRBC						
No vs Yes	1.216	0.551–3.213	0.651			
Intrinsic subtype HER2BC						
No vs Yes	0.536	0.030–2.460	0.494			
Intrinsic subtype TNBC						
No vs Yes	0.947	0.325–2.201	0.909			
Pathological axillary lymph node metastasis						
No metastasis vs Metastasis	0.945	0.385–2.002	0.891			
Lymph vascular invasion						
No vs Yes	1.673	0.888–3.076	0.109			
Hyperlipidemia						
No vs Yes	0.421	0.126–1.047	0.064	1.014	0.057–4.693	0.989
Multiple medicine types for hyperlipidemia						
No vs Yes	–	–	0.294			
Statins						
Non-user vs User	0.345	0.083–0.951	0.038	0.353	0.045–7.151	0.413
Lipophilic statins						
Non-user vs User	0.316	0.018–1.451	0.166			
Hydrophilic statins						
Non-user vs User	0.402	0.065–1.307	0.147			
Fibrate						
Non-user vs User	1.9701	0.111–9.059	0.545			
Nicotinic acid (tocopherol acetate)						
Non-user vs User	–	–	0.345			
Sterol absorption inhibitors (ezetimibe)						
Non-user vs User	–	–	0.409			

*HER2* Human epidermal growth factor receptor 2, *HRBC* Hormone receptor-positive breast cancer (ER+ and/or PgR+), *HER2BC* Human epidermal growth factor receptor 2-enriched breast cancer (ER-, PgR-, and HER2+), *TNBC* Triple negative breast cancer (ER-, PgR-, and HER2-), *CI* Confidence intervals

**Table 6:** Univariate and multivariate analysis with overall survival for cT1 breast cancer

Parameters	Univarite analysis			Multivarite analysis		
	Hazard ratio	95% CI	*p* value	Hazard ratio	95% CI	*p* value
Age at operation (years old)						
≤ 60 vs > 60	2.351	0.809–7.677	0.117			
Tumor size (mm)						
≤ 10.0 vs > 10.0	1.323	0.412–5.861	0.660			
Estrogen receptor						
Negative vs Positive	3.092	0.612–56.249	0.202			
Progesterone receptor						
Negative vs Positive	2.809	0.876–12.425	0.085	2.654	0.824–11.770	0.106
HER2						
Negative vs Positive	0.870	0.048–4.383	0.892			
Ki67						
≤ 20% vs > 20%	2.381	0.649–7.178	0.174			
Intrinsic subtype HRBC						
No vs Yes	2.596	0.515–47.194	0.292			
Intrinsic subtype HER2BC						
No vs Yes	–	–	0.243			
Intrinsic subtype TNBC						
No vs Yes	0.571	0.031–2.879	0.559			
Pathological axillary lymph node metastasis						
No metastasis vs Metastasis	0.858	0.133–3.150	0.838			
Lymph vascular invasion						
No vs Yes	1.324	0.406–3.839	0.621			
Hyperlipidemia						
No vs Yes	2.373	0.727–6.910	0.143			
Multiple medicine types for hyperlipidemia						
No vs Yes	6.109	0.335–31.184	0.169			
Statins						
Non-user vs User	2.605	0.798–7.578	0.107	2.425	0.742–7.057	0.134
Lipophilic statins						
Non-user vs User	2.287	0.355–8.449	0.328			
Hydrophilic statins						
Non-user vs User	2.253	0.508–7.247	0.251			
Fibrate						
Non-user vs User	7.765	0.424–40.367	0.131			
Nicotinic acid (tocopherol acetate)						
Non-user vs User	–	–	0.591			
Sterol absorption inhibitors (ezetimibe)						
Non-user vs User	–	–	0.672			

*HER2* Human epidermal growth factor receptor 2, *HRBC* Hormone receptor-positive breast cancer (ER+ and/or PgR+), *HER2BC* Human epidermal growth factor receptor 2-enriched breast cancer (ER-, PgR-, and HER2+). *TNBC* Triple negative breast cancer (ER-, PgR-, and HER2-), *CI* Confidence intervals

The prognoses were examined among the 607 patients who did not have macrometastases, and similar results were obtained. The median follow-up period was 1825 days (range, 54–4841 days). During that period, 35 patients (5.8%) experienced recurrence, two patients (0.3%) died from breast cancer, and 10 patients (1.6%) died from other causes. In the univariate analysis for DFS, tumor size (*p* = 0.084, HR: 1.888) and PgR status (*p* = 0.032, HR: 1.977) affected prognosis, whereas the use of hyperlipidemic drugs did not (Online Resource Supplementary Table 2). On the other hand, in the univariate analysis for RFS, tumor size (*p* = 0.036, HR: 2.493) and PgR status (*p* = 0.043, HR: 2.064) affected the prognosis (Online Resource Supplementary Table 3). The analysis revealed that statin use (*p* = 0.096, HR: 0.411) tended to affect prognosis, but this did not reach statistical significance (Online Resource Supplementary Fig. 1). In the univariate analysis for OS, statin use (*p* = 0.047, HR: 3.460) was poor prognostic factor; in the multivariate analysis, no independent factors were found (Online Resource Supplementary Table 4).

## Discussion

In a study examining the correlation between lymph node metastasis and clinicopathological features among 91,364 patients with T1 breast cancer using information from the “Surveillance, Epidemiology, and End Results Program (SEER)” study, age, race, primary site, tumor size, and ER, PgR, and HER2 status were influencing factors [38]. Tumor size and lymphovascular invasion are cited as risk factors for lymph node metastasis in most studies involving sentinel lymph node biopsy [39–46]. This result also shows that tumor size and lymphovascular invasion were strongly correlated with lymph node metastasis, which is consistent with previously reported results. Among the investigated factors, this study showed that the use of lipophilic statins may suppress lymph node metastasis. In preclinical studies, statins have been shown to exhibit anti-proliferative on cancer by being associated with mechanisms that drive cell cycle disruption in cancer cells [13–17]. Many studies have investigated the effects of factors capable of suppressing the risk of breast cancer and its recurrence, and there have also been some reports examining the effects of statins on suppressing the progression of breast cancer. For example, when examining the correlation between statin use and clinicopathological factors at the time of diagnosis in about 2000 and 3000 breast cancer patients, respectively, the rates of diagnosis for breast cancer with high pathological malignancy and for highly advanced breast cancer were significantly lower in statin users than in non-users [27, 28]. In addition, a study of approximately 130,000 postmenopausal women conducted by the Women’s Health Initiative reported that the use of lipophilic statins reduced the rate of diagnosis of highly advanced breast cancer [29]. However, the opportunity for patient consultation is likely to strongly influence these results. On the other hand, in this study, the tumor size based on the TNM classification was used as a condition for examination; this methodology is different from that of previous reports. In preclinical studies, anti-invasive properties have also been reported [18–22], as have metastasis-suppressing effects [23–26]. This study demonstrates the possibility of suppressing lymph node metastasis in clinical practice, which could improve prognosis.

Based on many results from preclinical studies, it is expected that statins should suppress the risk of breast cancer and its recurrence. However, in clinical practice, contradictory results have been reported regarding the suppressing effect of statins on breast cancer risk [6, 47, 48]. One reports have discussed why prospective studies with statins have not yielded the expected results [49]. On the other hand, many studies have reported that statins reduce the risk of breast cancer recurrence, and some groups have reported that only lipophilic statins are effective, not hydrophilic statins [4, 5, 7–10, 12, 42]. A report indicated that effects may vary considerably among lipophilic statins [49]. The classification of statins in this study was the same as that used in a meta-analysis that examined the correlation between statin type and breast cancer prognosis [50]. In this study, statins reduced OS in patients without lymph node metastases. However, this result is likely due to the fact that only two patients (0.3%) died from breast cancer and 10 patients (1.6%) died from other causes. Breast-cancer-specific survival could not be examined due to the low numbers of breast cancer-related deaths; therefore, the results pertaining to OS in this study should be considered for reference. However, statin use tended to prolong the RFS period, instead of the DFS period. Regarding this result, the event point was narrowed down to the day of recurrence / death from breast cancer, suggesting that statins may have a positive effect on the treatment of early-stage breast cancer.

This study has some limitations that should be considered. First, patients receiving preoperative chemotherapy were excluded, as the evaluation of axillary lymph node metastasis is uncertain based on diagnostic imaging alone. Since it is known that the therapeutic effect of preoperative chemotherapy is a predictor of prognosis in HER2-positive breast cancer and TNBC [51–54], preoperative chemotherapy is actively performed for those types of breast cancer. The number of patients with HER2-positive breast cancer and TNBC was low, which could have been a source of bias in this study. In addition, statin was correlated with age, although age itself had no clear effect on axillary lymph node metastasis or prognosis in this study, it may have a significant effect. Moreover, one of the limitations was the exclusion of cases involving a primary lesion of 20 mm or less, accompanied by advanced regional lymph node metastasis or distant metastasis. Another limitation was that the duration of oral treatment for hyperlipidemia was unknown for each patient. However, clinical data, rather than in vivo or in vitro data, suggest that lipophilic statins may suppress breast cancer metastasis to lymph nodes. Furthermore, it was suggested that statins may suppress postoperative recurrence. Regarding the examination and treatment of axillary lymph nodes, in recent years, even sentinel lymph node biopsy has been deemed an overly invasive procedure for early-stage breast cancer cases, so clinical trials are underway to omit sentinel lymph node biopsies from the protocols for cN0 breast cancer cases assessed using US [55, 56]. It is also possible that lipophilic statins may have affected the results of these clinical trials. Regarding the prognosis, some studies have reported that even if statins are administered after the diagnosis of breast cancer, they may suppress the recurrence of breast cancer [4, 5, 7, 9, 10, 30]. Especially in ER-positive breast cancer, the effects driving the suppression of the risk of recurrence are well-recognized [5, 30]. The fact that the prognosis was affected in this study may have been due to the fact that ER-positive breast cancer patients accounted for the majority of the cases. This study suggests the possibility of improving the prognosis of breast cancer patients through treatment with statins.

## Conclusions

In patients with cT1 breast cancer, the results suggest that oral statin therapy may contribute to favorable outcomes.

## Supplementary Information

**Additional file 1: Supplementary Fig. 1.** Kaplan–Meier method comparing recurrence-free survival (RFS) and overall survival (OS) by statin or lipophilic statin in patients without lymph node metastasis. There was no significant difference in RFS due to statin (A) and lipophilic statin (B). However, statin user had poor OS (*p* = 0.025, log-rank) (C). No impact on OS was found in lipophilic statin(D).

**Additional file 2: Supplementary Table 1.** Univariate and multivariate analysis with disease-free survival for cT1 breast cancer. **Supplementary Table 2.** Univariate and multivariate analysis with disease-free survival for cT1 breast cancer with no axillary lymph node metastasis pathologically. **Supplementary Table 3.** Univariate and multivariate analysis with recurrence-free survival for cT1 breast cancer with no axillary lymph node metastasis pathologically. **Supplementary Table 4.** Univariate and multivariate analysis with overall survival for cT1 breast cancer with no axillary lymph node metastasis pathologically.

## Data Availability

The datasets used and/or analysed during the current study available from the corresponding author on reasonable request.

## Abbreviations

CI: Confidence intervals
CNB: Core needle biopsy
CT: Computed tomography
DFS: Disease-free survival
ER: Estrogen receptor
HER2: Human epidermal growth factor receptor 2
HR: Hormone receptor
OR: Odds ratio
OS: Overall survival
PgR: Progesterone receptor
RFS: Recurrence-free survival
TNBC: Triple-negative breast cancer
US: Ultrasonography
