# Supplementary figures and images for: Impact of oral statin therapy on clinical outcomes in patients with cT1 breast cancer
Source: BMC Cancer. 2023 Mar 9;23:224. doi: 10.1186/s12885-023-10631-w (PMC9999569; doi:10.1186/s12885-023-10631-w)

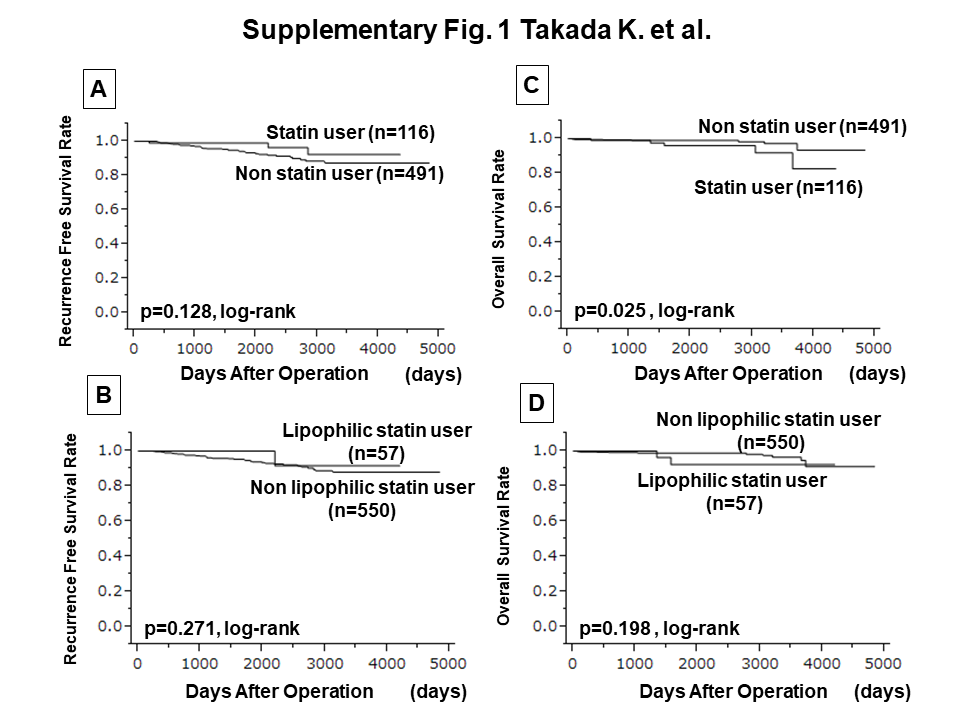

Supplement: Supplementary file 1 — Additional file 1: Supplementary Fig. 1. Kaplan–Meier method comparing recurrence-free survival (RFS) and overall survival (OS) by statin or lipophilic statin in patients without lymph node metastasis. There was no significant difference in RFS due to statin (A) and lipophilic statin (B). However, statin user had poor OS (p = 0.025, log-rank) (C). No impact on OS was found in lipophilic statin(D). [file 12885_2023_10631_MOESM1_ESM.tif]
