# Supplementary material for: Impact of oral statin therapy on clinical outcomes in patients with cT1 breast cancer
Source: BMC Cancer. 2023 Mar 9;23:224. doi: 10.1186/s12885-023-10631-w (PMC9999569; doi:10.1186/s12885-023-10631-w)
Supplement: Supplementary file 2 — Additional file 2: Supplementary Table 1. Univariate and multivariate analysis with disease-free survival for cT1 breast cancer. Supplementary Table 2. Univariate and multivariate analysis with disease-free survival for cT1 breast cancer with no axillary lymph node metastasis pathologically. Supplementary Table 3. Univariate and multivariate analysis with recurrence-free survival for cT1 breast cancer with no axillary lymph node metastasis pathologically. Supplementary Table 4. Univariate and multivariate analysis with overall survival for cT1 breast cancer with no axillary lymph node metastasis pathologically. [file 12885_2023_10631_MOESM2_ESM.docx]

**Supplementary Table 1. Univariate and multivariate analysis with disease-free survival for cT1 breast cancer**

|  | Univarite analysis | | |  | Multivarite analysis | | |
| --- | --- | --- | --- | --- | --- | --- | --- |
| Parameters | Hazard ratio | 95 % CI | *p* value |  | Hazard ratio | 95 % CI | *p* value |
| Age at operation (years old)  ≤ 60 vs > 60 | 0.694 | 0.367-1.268 | 0.238 |  |  |  |  |
| Tumor size (mm)  ≤ 10.0 vs > 10.0 | 2.902 | 1.255-8.423 | 0.011 |  | 2.620 | 1.117-7.668 | 0.025 |
| Estrogen receptor  Negative vs Positive | 1.036 | 0.506-2.405 | 0.928 |  |  |  |  |
| Progesterone receptor  Negative vs Positive | 1.463 | 0.797-2.803 | 0.223 |  |  |  |  |
| HER2  Negative vs Positive | 0.837 | 0.203-2.304 | 0.761 |  |  |  |  |
| Ki67  ≤20 % vs >20 % | 0.919 | 0.348-2.020 | 0.846 |  |  |  |  |
| Intrinsic subtype HRBC  No vs Yes | 1.059 | 0.502-2.598 | 0.889 |  |  |  |  |
| Intrinsic subtype HER2BC  No vs Yes | 0.514 | 0.029-2.355 | 0.462 |  |  |  |  |
| Intrinsic subtype TNBC  No vs Yes | 1.120 | 0.425-2.462 | 0.800 |  |  |  |  |
| Pathological axillary lymph node metastasis  No metastasis vs Metastasis | 1.056 | 0.456-2.158 | 0.889 |  |  |  |  |
| Lymph vascular invasion  No vs Yes | 1.712 | 0.924-3.104 | 0.086 |  | 1.441 | 0.772-2.633 | 0.246 |
| Hyperlipidemia  No vs Yes | 0.399 | 0.120-0.990 | 0.047 |  | 0.953 | 0.054-4.399 | 0.962 |
| Multiple medicine types for hyperlipidemia  No vs Yes | - | - | 0.284 |  |  |  |  |
| Statins  Non-user vs User | 0.328 | 0.079-0.901 | 0.028 |  | 0.372 | 0.047-7.535 | 0.435 |
| Lipophilic statins  Non-user vs User | 0.298 | 0.017-1.368 | 0.142 |  |  |  |  |
| Hydrophilic statins  Non-user vs User | 0.385 | 0.063-1.248 | 0.125 |  |  |  |  |
| Fibrate  Non-user vs User | 1.866 | 0.105-8.559 | 0.575 |  |  |  |  |
| Nicotinic acid (tocopherol acetate)  Non-user vs User | - | - | 0.335 |  |  |  |  |
| Sterol absorption inhibitors (ezetimibe)  Non-user vs User | - | - | 0.397 |  |  |  |  |

HER2: human epidermal growth factor receptor 2. HRBC: hormone receptor-positive breast cancer (ER+ and/or PgR+). HER2BC: human epidermal growth factor receptor 2-enriched breast cancer (ER-, PgR-, and HER2+). TNBC: triple negative breast cancer (ER-, PgR-, and HER2-). CI, confidence intervals.

**Supplementary Table 2. Univariate and multivariate analysis with disease-free survival for cT1 breast cancer with no axillary lymph node metastasis pathologically.**

|  | Univarite analysis | | |  | Multivarite analysis | | |
| --- | --- | --- | --- | --- | --- | --- | --- |
| Parameters | Hazard ratio | 95 % CI | *p* value |  | Hazard ratio | 95 % CI | *p* value |
| Age at operation (years old)  ≤ 60 vs > 60 | 0.840 | 0.453-1.523 | 0.569 |  |  |  |  |
| Tumor size (mm)  ≤ 10.0 vs > 10.0 | 1.888 | 0.924-4.377 | 0.084 |  | 1.898 | 0.929-4.399 | 0.081 |
| Estrogen receptor  Negative vs Positive | 1.065 | 0.520-2.472 | 0.872 |  |  |  |  |
| Progesterone receptor  Negative vs Positive | 1.977 | 1.058-3.913 | 0.032 |  | 1.984 | 1.062-3.928 | 0.031 |
| HER2  Negative vs Positive | 0.805 | 0.195-2.215 | 0.709 |  |  |  |  |
| Ki67  ≤20 % vs >20 % | 1.006 | 0.410-2.123 | 0.988 |  |  |  |  |
| Intrinsic subtype HR+BC  No vs Yes | 1.131 | 0.536-2.775 | 0.763 |  |  |  |  |
| Intrinsic subtype HR-HER2+BC  No vs Yes | 0.522 | 0.029-2.391 | 0.474 |  |  |  |  |
| Intrinsic subtype TNBC  No vs Yes | 1.025 | 0.389-2.254 | 0.956 |  |  |  |  |
| Lymph vascular invasion  No vs Yes | 1.528 | 0.786-2.828 | 0.203 |  |  |  |  |
| Hyperlipidemia  No vs Yes | 0.870 | 0.376-1.777 | 0.718 |  |  |  |  |
| Multiple medicine types for hyperlipidemia  No vs Yes | 2.399 | 0.135-11.009 | 0.448 |  |  |  |  |
| Statins  Non-user vs User | 0.829 | 0.339-1.748 | 0.643 |  |  |  |  |
| Lipophilic statins  Non-user vs User | 0.850 | 0.206-2.341 | 0.782 |  |  |  |  |
| Hydrophilic statins  Non-user vs User | 0.839 | 0.252-2.081 | 0.731 |  |  |  |  |
| Fibrate  Non-user vs User | 3.610 | 0.588-11.734 | 0.139 |  |  |  |  |
| Nicotinic acid (tocopherol acetate)  Non-user vs User | - | - | 0.422 |  |  |  |  |
| Sterol absorption inhibitors (ezetimibe)  Non-user vs User | - | - | 0.433 |  |  |  |  |

HER2: human epidermal growth factor receptor 2. HR+BC: hormone receptor-positive breast cancer (ER+ and/or PgR+). HR-HER2+BC: human epidermal growth factor receptor 2-enriched breast cancer (ER-, PgR-, and HER2+). TNBC: triple negative breast cancer (ER-, PgR-, and HER2-). CI, confidence intervals.

**Supplementary table 3. Univariate and multivariate analysis with recurrence-free survival for cT1 breast cancer with no axillary lymph node metastasis pathologically.**

|  | Univarite analysis | | |  | Multivarite analysis | | |
| --- | --- | --- | --- | --- | --- | --- | --- |
| Parameters | Hazard ratio | 95 % CI | *p* value |  | Hazard ratio | 95 % CI | *p* value |
| Age at operation (years old)  ≤ 60 vs > 60 | 0.556 | 0.262-1.108 | 0.096 |  | 0.690 | 0.312-1.427 | 0.325 |
| Tumor size (mm)  ≤ 10.0 vs > 10.0 | 2.493 | 1.055-7.320 | 0.036 |  | 2.282 | 0.952-6.760 | 0.066 |
| Estrogen receptor  Negative vs Positive | 0.958 | 0.441-2.390 | 0.920 |  |  |  |  |
| Progesterone receptor  Negative vs Positive | 2.064 | 1.021-4.508 | 0.043 |  | 2.065 | 1.019-4.525 | 0.044 |
| HER2  Negative vs Positive | 0.674 | 0.109-2.222 | 0.566 |  |  |  |  |
| Ki67  ≤20 % vs >20 % | 0.507 | 0.122-1.418 | 0.217 |  |  |  |  |
| Intrinsic subtype HR+BC  No vs Yes | 1.046 | 0.464-2.796 | 0.920 |  |  |  |  |
| Intrinsic subtype HR-HER2+BC  No vs Yes | 0.657 | 0.037-3.046 | 0.658 |  |  |  |  |
| Intrinsic subtype TNBC  No vs Yes | 1.068 | 0.363-2.528 | 0.893 |  |  |  |  |
| Lymph vascular invasion  No vs Yes | 1.921 | 0.941-3.764 | 0.072 |  | 1.634 | 0.794-3.243 | 0.176 |
| Hyperlipidemia  No vs Yes | 0.505 | 0.150-1.277 | 0.161 |  |  |  |  |
| Multiple medicine types for hyperlipidemia  No vs Yes | - | - | 0.416 |  |  |  |  |
| Statins  Non-user vs User | 0.411 | 0.099-1.148 | 0.096 |  | 0.517 | 0.119-1.574 | 0.267 |
| Lipophilic statins  Non-user vs User | 0.345 | 0.019-1.599 | 0.210 |  |  |  |  |
| Hydrophilic statins  Non-user vs User | 0.505 | 0.082-1.662 | 0.298 |  |  |  |  |
| Fibrate  Non-user vs User | 2.248 | 0.126-10.424 | 0.479 |  |  |  |  |
| Nicotinic acid (tocopherol acetate)  Non-user vs User | - | - | 0.471 |  |  |  |  |
| Sterol absorption inhibitors (ezetimibe)  Non-user vs User | - | - | 0.487 |  |  |  |  |

HER2: human epidermal growth factor receptor 2. HR+BC: hormone receptor-positive breast cancer (ER+ and/or PgR+). HR-HER2+BC: human epidermal growth factor receptor 2-enriched breast cancer (ER-, PgR-, and HER2+). TNBC: triple negative breast cancer (ER-, PgR-, and HER2-). CI, confidence intervals.

**Supplementary Table 4. Univariate and multivariate analysis with overall survival for cT1 breast cancer with no axillary lymph node metastasis pathologically.**

|  | Univarite analysis | | |  | Multivarite analysis | | |
| --- | --- | --- | --- | --- | --- | --- | --- |
| Parameters | Hazard ratio | 95 % CI | *p* value |  | Hazard ratio | 95 % CI | *p* value |
| Age at operation (years old)  ≤ 60 vs > 60 | 2.726 | 0.853-10.279 | 0.091 |  | 2.057 | 0.577-8.300 | 0.269 |
| Tumor size (mm)  ≤ 10.0 vs > 10.0 | 1.202 | 0.357-5.435 | 0.780 |  |  |  |  |
| Estrogen receptor  Negative vs Positive | 2.716 | 0.525-49.718 | 0.273 |  |  |  |  |
| Progesterone receptor  Negative vs Positive | 2.449 | 0.731-11.042 | 0.153 |  |  |  |  |
| HER2  Negative vs Positive | 0.985 | 0.054-5.074 | 0.988 |  |  |  |  |
| Ki67  ≤20 % vs >20 % | 2.767 | 0.736-8.829 | 0.123 |  |  |  |  |
| Intrinsic subtype HR+BC  No vs Yes | 2.324 | 0.461-42.507 | 0.364 |  |  |  |  |
| Intrinsic subtype HR-HER2+BC  No vs Yes | - | - | 0.273 |  |  |  |  |
| Intrinsic subtype TNBC  No vs Yes | 0.634 | 0.035-3.272 | 0.643 |  |  |  |  |
| Lymph vascular invasion  No vs Yes | 1.033 | 0.229-3.467 | 0.962 |  |  |  |  |
| Hyperlipidemia  No vs Yes | 3.160 | 0.927-10.035 | 0.065 |  | - | - | 0.585 |
| Multiple medicine types for hyperlipidemia  No vs Yes | 9.095 | 0.494-47.938 | 0.110 |  |  |  |  |
| Statins  Non-user vs User | 3.460 | 1.016-10.963 | 0.047 |  | - | - | 0.387 |
| Lipophilic statins  Non-user vs User | 2.627 | 0.401-10.097 | 0.266 |  |  |  |  |
| Hydrophilic statins  Non-user vs User | 2.967 | 0.655-10.056 | 0.142 |  |  |  |  |
| Fibrate  Non-user vs User | 8.932 | 0.483-48.060 | 0.113 |  |  |  |  |
| Nicotinic acid (tocopherol acetate)  Non-user vs User | - | - | 0.669 |  |  |  |  |
| Sterol absorption inhibitors (ezetimibe)  Non-user vs User | - | - | 0.708 |  |  |  |  |

HER2: human epidermal growth factor receptor 2. HR+BC: hormone receptor-positive breast cancer (ER+ and/or PgR+). HR-HER2+BC: human epidermal growth factor receptor 2-enriched breast cancer (ER-, PgR-, and HER2+). TNBC: triple negative breast cancer (ER-, PgR-, and HER2-). CI, confidence intervals.
